# Impact of chemoradiotherapy on the survival of unresectable locally advanced pancreatic cancer: a retrospective cohort analysis

**DOI:** 10.1186/s12876-023-02739-x

**Published:** 2023-04-05

**Authors:** Zi-Meng Wang, Hong-Bin Ma, Yan Meng

**Affiliations:** Department of Radiation Oncology, Third Affiliated Hospital of Naval Medical University (Eastern Hepatobiliary Surgery Hospital), Shanghai, 200438 China

**Keywords:** Unresectable, Locally advanced pancreatic cancer, Chemoradiotherapy, Survival, SEER program database

## Abstract

**Background:**

The role of chemoradiotherapy in unresectable locally advanced pancreatic cancer is still unclear.

**Methods:**

Data from patients with unresectable locally advanced pancreatic cancer were extracted from the Surveillance, Epidemiology, and End Results Program database. Univariate and multivariate Cox regression analyses were conducted to identify the independent prognostic factors of survival. Propensity score matching was carried out to minimize the interference of confounding factors. Subgroup analysis was performed to screen the characteristics of patients who would benefit from chemoradiotherapy.

**Results:**

A total of 5002 patients with unresectable locally advanced pancreatic cancer were included. Among them, 2423 (48.4%) received chemotherapy, and 2579 (51.6%) received chemoradiotherapy. The median overall survival of all patients was 11 months. Multivariate Cox analysis showed that age (*p* < 0.001), marital status (*p* < 0.001), tumor size (*p* = 0.001), N stage (*p* = 0.015) and radiotherapy (*p* < 0.001) were independent prognostic factors of survival. Both before (HR, 0.817; 95% CI, 0.769–0.868; *p* < 0.001) and after (HR, 0.904; 95% CI, 0.876–0.933; *p* < 0.001) propensity score matching, chemoradiotherapy significantly improved the median overall survival of patients from 10 to 12 months. Subgroup analysis showed that chemoradiotherapy was significantly associated with improved survival regardless of sex, primary site or N stage. In addition, the following subgroups all significantly benefited from chemoradiotherapy: age ≥ 50 years, not divorced, grade 2–4, tumor size > 2 cm, adenocarcinoma, mucinous adenocarcinoma and white race.

**Conclusions:**

Chemoradiotherapy is highly recommended for patients with unresectable locally advanced pancreatic cancer.

**Supplementary Information:**

The online version contains supplementary material available at 10.1186/s12876-023-02739-x.

## Background

Pancreatic cancer is an extremely fatal malignancy with a similar number of annual new cancer cases and deaths [1–3]. It can be classified into four types according to tumor resectability: resectable, borderline resectable, locally advanced, and metastatic [1]. Surgical treatment is the only potential curative strategy. However, due to the absence of specific symptoms, early detection is difficult, and only approximately 15%-20% of patients have the opportunity to undergo surgery by the time they are diagnosed [1, 4]. Locally advanced pancreatic cancer is a nonmetastatic type that cannot be surgically resected owing to the invasion of vascular structures. As a result, it is almost impossible to cure and has a poor prognosis with an overall survival between 9 and 13 months[5].

Since surgery cannot be performed, chemotherapy and radiotherapy seem to be the remaining options for unresectable locally advanced pancreatic cancer (ULAPC). Many studies have demonstrated the importance of chemotherapy for improving the survival of ULAPC patients [6–8]. No consensus has been reached regarding the use of radiotherapy. Among relevant randomized controlled studies retrieved from PubMed [9–13], some studies [10, 12] have found that chemoradiotherapy (CRT) is superior to chemotherapy (CT), while others [9, 11, 13] found no survival benefits from chemoradiotherapy.

The Surveillance, Epidemiology, and End Results Program (SEER) database collects data on cancer cases from various locations and sources throughout the United States, which provides useful information for clinical cancer research [14]. Accordingly, in this paper, we extracted massive historical statistics from the SEER database to retrospectively verify the efficacy of chemoradiotherapy on ULAPC.

## Methods

### Patient selection

We used SEER software (version 8.3.9) to extract data from the SEER*Stat Database: Incidence—SEER 18 Regs Custom Data (with additional treatment fields), Nov 2018 Sub (1975–2016 varying).

**Fig. 1:**
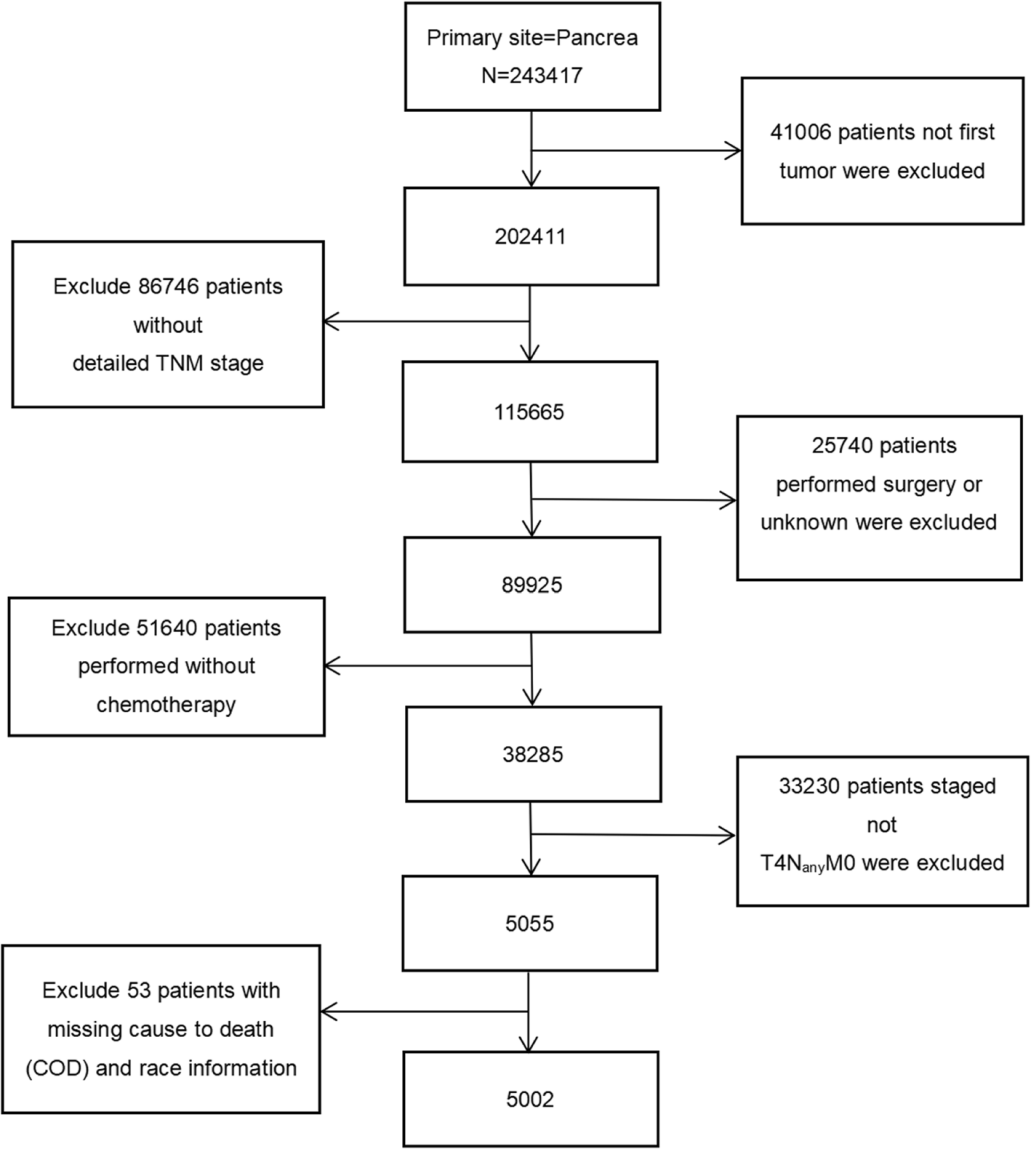
The flowchart of the selection process for the study cohort

To obtain as many cases as possible, we used the keyword “primary site-labeled = C25.9 pancreas” to extract sufficient data and screen carefully, as shown in Fig. 1. Our exclusion criteria were as follows: (1) not the patients’ first primary tumor; (2) patients without detailed TNM stage; (3) patients received surgical treatment or unknown; (4) patients did not receive chemotherapy; (5) patients staged not T_4_N_any_M_0_ (AJCC 6/7th stage III); and (6) patients with missing cause to death (COD) and race information. Notably, all selected cases were malignant (ICD-O-3), and the most common pathological types were as follows: 8140/3: adenocarcinoma, 8500/3: infiltrating duct carcinoma, 8480/3: mucinous adenocarcinoma and 8246/3: neuroendocrine carcinoma.

We extracted detailed and critical variables from each record. Demographic features such as age, sex, race and marital status and clinicopathological characteristics such as primary site, pathological type, grade, tumor size and N stage were all included. Specifically, tumor size was classified according to the AJCC 8th stage.

### Statistical analysis

R software (version 4.0.5) was employed to perform all of the statistical analyses and diagrams. The “Table one” package was used to compare the differences between the two sets of variables. The “Survival” package was used for survival analysis and Kaplan–Meier curve drawing. Univariate and multivariate Cox proportional hazard regression analyses were conducted on all patients using the “coxph” function. To ensure consistency between the two groups of variables, we used the “MatchIt” package to perform propensity score matching (PSM) analysis. PSM was carried out using the 1:1 nearest test with a caliper value of 0.05. The “Forestplot” package was utilized to depict forest plots based on subgroup analysis. A significant *p* value was set at 0.05.

## Results

### Patient characteristics

A total of 5002 patients with unresectable locally advanced pancreatic cancer were included. Among these patients, 2423 (48.4%) received chemotherapy, and 2579 (51.6%) received chemoradiotherapy. A majority of patients were aged 50 to 74 years (71.6%) and 51.9% were men. Most patients (67.5%) had no invasion of the lymph nodes, and 60.0% were married. Adenocarcinoma (82.9%) and infiltrating duct carcinoma (7.2%) accounted for 91.1% of all pathologic types. Most of the tumors occurred in the head of the pancreas (55.1%), and 42.5% were larger than 4 cm in size. When comparing patients receiving chemotherapy with those receiving chemoradiotherapy, there was a significant difference in age (*p* = 0.004), primary site (*p* = 0.031), pathological type (*p* = 0.001), and N stage (*p* < 0.001), as shown in Table 1.

**Table 1:** Demographic and clinicopathological characteristics of patients receiving chemotherapy alone and chemoradiotherapy

Characteristic	Chemotherapy (2423)	Chemoradiotherapy (2579)	*P* value
Age			0.004
25–49	205 ( 8.46%)	191 ( 7.41%)	
50–74	1767 (72.93%)	1813 (70.30%)	
≥ 75	451 (18.61%)	575 (22.30%)	
Sex			0.444
Male	1244 (51.34%)	1353 (52.46%)	
Female	1179 (48.66%)	1226 (47.54%)	
Race			0.911
White	1887 (77.88%)	2012 (78.01%)	
Black	311 (12.84%)	336 (13.03%)	
Other	225 ( 9.29%)	231 ( 8.96%)	
Marital status			0.694
Married	1469 (60.63%)	1534 (59.48%)	
Single	269 (11.10%)	290 (11.24%)	
Divorced	287 (11.84%)	333 (12.91%)	
Other	398 (16.43%)	422 (16.36%)	
Primary Site			0.031
Head	1377 (56.83%)	1377 (53.39%)	
Body/tail	562 (23.19%)	621 (24.08%)	
Other	484 (19.98%)	581 (22.53%)	
Pathological type			0.001
8140/3	2045 (84.40%)	2104 (81.58%)	
8500/3	183 ( 7.55%)	178 ( 6.90%)	
8480/3	42 ( 1.73%)	63 ( 2.44%)	
8246/3	11 ( 0.45%)	29 ( 1.12%)	
Other	142 ( 5.86%)	205 ( 7.95%)	
Grade			0.483
G1	109 ( 4.50%)	119 ( 4.61%)	
G2	282 (11.64%)	263 (10.20%)	
G3	285 (11.76%)	288 (11.17%)	
G4	19 ( 0.78%)	22 ( 0.85%)	
Gx	1728 (71.32%)	1887 (73.17%)	
Tumor size			0.426
≤ 2 cm	89 ( 3.67%)	94 ( 3.64%)	
2-4 cm	1054 (43.50%)	1064 (41.26%)	
> 4 cm	1010 (41.68%)	1114 (43.20%)	
Unknown	270 (11.14%)	307 (11.90%)	
Nstage			< 0.001
N0	1406 (58.03%)	1329 (51.53%)	
N1	857 (35.37%)	986 (38.23%)	
Nx	160 ( 6.60%)	264 (10.24%)	

8140/3, adenocarcinoma; 8500/3, infiltrating duct carcinoma; 8480/3, mucinous adenocarcinoma; 8246/3, neuroendocrine carcinoma

### Survival analysis of all patients

**Fig. 2:**
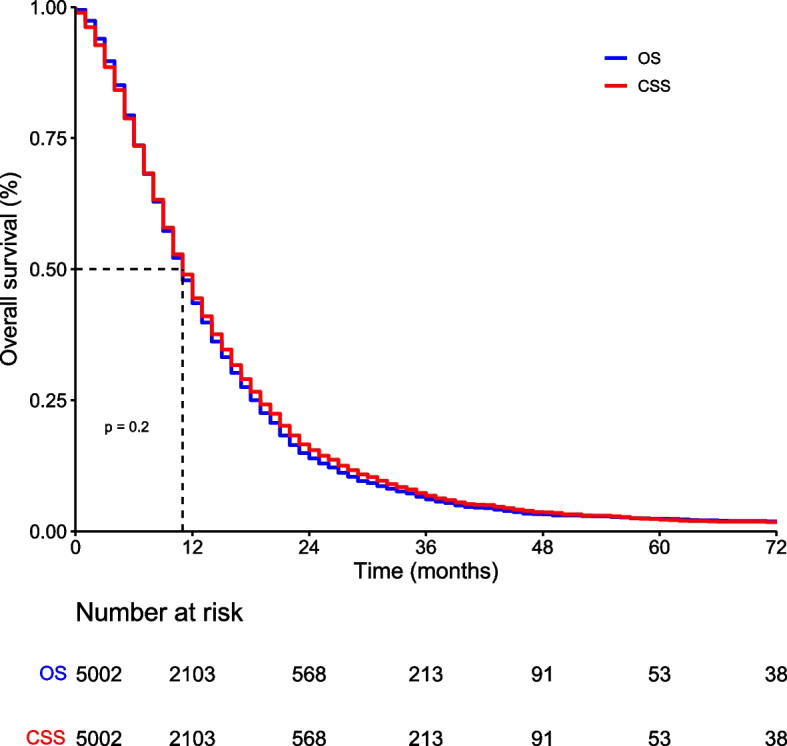
Kaplan–Meier curves of overall survival (OS) and cancer-specific survival (CSS) of all patients

As shown in Fig. 2, the Kaplan–Meier curves of overall survival (OS) and cancer-specific survival (CSS) almost overlapped (*p* = 0.2). The median OS and CSS of all patients were both 11 months. The 1-, 2-, and 3-year OS rates were 43.5%, 13.9%, and 6.1%, respectively. The 1-, 2-, and 3-year CSS rates were 44.4%, 15.5%, and 6.8%, respectively. Of all 4262 deaths, cancer-specific deaths (4150) accounted for 97.4%, while other cause-specific deaths (112) accounted for only 2.6%. Therefore, in the following analyses, we used OS as the study endpoint.

**Fig. 3:**
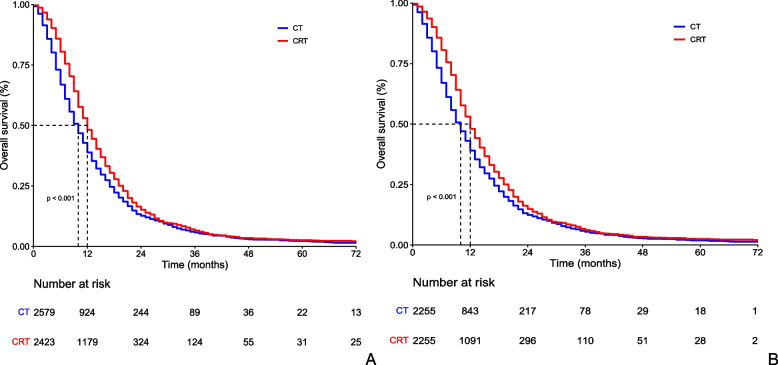
Comparsion of overall survival between chemoradiotherapy and chemotherapy. **A** Before propensity score matching; **B** After propensity score matching

Table 2 summarizes the results of univariate and multivariate Cox regression analyses. According to the results of univariate analysis, age (*p* < 0.001), marital status (*p* < 0.001), tumor size (*p* = 0.001), N stage (*p* = 0.015) and radiation (*p* < 0.001) were correlated with survival. These variables were then incorporated into multivariate analysis. The results of multivariate analysis showed that all the variables above were still statistically significant. Age ≥ 75 years, single, tumor larger than 4 cm, regional lymph node metastasis and no radiotherapy indicated worse survival. Moreover, before propensity score matching, chemoradiotherapy showed obvious survival benefits compared with chemotherapy [median OS: 12 months vs. 10 months (HR, 0.817; 95% CI, 0.769–0.868; *p* < 0.001)] (Fig. 3A). Figs. S1 and S2 show the forest plots based on the hazard ratio (HR) and overall survival rates before PSM analysis.

**Table 2:** Univariate and multivariate analysis of overall survival

Characteristic	HR	CI	*p*	HR	CI	*p*
Age			< 0.001			< 0.001
25–49	1					
50–74	0.961	0.860–1.074	0.483	0.976	0.873–1.091	0.666
≥ 75	1.315	1.161–1.489	< 0.001	1.319	1.161–1.497	< 0.001
Sex			0.404			
Male	1					
Female	0.975	0.918—1.035				
Race			0.683			
White	1					
Black	0.982	0.897–1.075	0.694			
Other	0.985	0.885–1.096	0.779			
Marital status			< 0.001			0.004
Married	1					
Single	1.125	1.021–1.240	0.018	1.148	1.041–1.266	0.006
Divorced	1.046	0.952–1.149	0.348	1.062	0.966–1.167	0.213
Other	1.175	1.081–1.277	< 0.001	1.104	1.014–1.202	0.023
Primary Site			0.771			
Head	1					
Body/tail	0.959	0.891–1.034	0.278			
Other	0.999	0.925–1.079	0.979			
Pathological type			0.813			
8140/3	1					
8500/3	0.972	0.866–1.093	0.638			
8480/3	0.893	0.725–1.102	0.293			
8246/3	0.321	0.221- 0.466	< 0.001			
Other	1.163	1.035–1.307	0.011			
Grade			0.484			
G1	1					
G2	1.251	1.059–1.477	0.008			
G3	1.776	1.505–2.097	< 0.001			
G4	1.931	1.373–2.097	< 0.001			
Gx	1.300	1.124–1.503	< 0.001			
Tumor size			0.001			< 0.001
≤ 2 cm	1					
2-4 cm	1.066	0.908–1.252	0.434	1.063	0.905–1.249	0.455
> 4 cm	1.176	1.001–1.381	0.048	1.192	1.015–1.401	0.032
Unknown	1.179	0.988–1.406	0.067	1.183	0.992–1.412	0.061
Nstage			0.015			0.045
N0	1					
N1	1.092	1.024–1.164	0.007	1.083	1.016–1.155	0.015
Nx	1.071	0.947–1.218	0.291	1.047	0.921–1.191	0.482
Raditaion			< 0.001			< 0.001
Yes	1			1		
No	1.224	1.153—1.300		1.206	1.135—1.281	

8140/3, adenocarcinoma; 8500/3, infiltrating duct carcinoma; 8480/3, mucinous adenocarcinoma; 8246/3, neuroendocrine carcinoma

### Survival analysis after propensity score matching

To control the interference of confounding factors and more accurately assess the efficacy of chemoradiotherapy in ULAPC patients, we used 1:1 PSM analysis to minimize bias. As shown in Table 3, there was no significant difference in the characteristics of the two groups after PSM analysis. In the matched group, chemoradiotherapy still had stronger survival than chemotherapy [median OS: 12 months vs. 10 months (HR, 0.904; 95% CI, 0.876–0.933; *p* < 0.001)] (Fig. 3B).

**Table 3:** Demographic and clinicopathological characteristics of patients receiving chemotherapy alone and chemoradiotherapy after propensity score matching

Characteristic	Chemotherapy(2255)	Chemoradiotherapy(2255)	*P* value
Age			0.867
25–49	171 ( 7.6%)	175 ( 7.8%)	
50–74	1639 (72.7%)	1623 (72.0%)	
≥ 75	445 (19.7%)	457 (20.3%)	
Sex			0.835
Male	1169(51.8%)	1177(52.2%)	
Female	1086 (48.2%)	1078 (47.8%)	
Race			0.517
White	1733 (76.9%)	1762 (78.1%)	
Black	300 (13.3%)	291 (12.9%)	
Other	222 ( 9.8%)	202 ( 9.0%)	
Marital status			0.352
Married	1356 (60.1%)	1377 (61.1%)	
Single	230 (10.2%)	242 (10.7%)	
Divorced	273 (12.1%)	284 (12.6%)	
Other	396 (17.6%)	352 (15.6%)	
Primary Site			0.498
Head	1270 (56.3%)	1232 (54.6%)	
Body/tail	515 (22.8%)	542 (24.0%)	
Other	470 (20.8%)	481 (21.3%)	
Pathological type			0.379
8140/3	1892 (83.9%)	1905 (84.5%)	
8500/3	169 ( 7.5%)	152 ( 6.7%)	
8480/3	42 ( 1.9%)	47 ( 2.1%)	
8246/3	11 ( 0.5%)	20 ( 0.9%)	
Other	141(6.3%)	131(5.8%)	
Grade			0.702
G1	101 ( 4.5%)	109 ( 4.8%)	
G2	245 (10.9%)	238 (10.6%)	
G3	279 (12.4%)	251 (11.1%)	
G4	18 ( 0.8%)	20 ( 0.9%)	
Gx	1612(71.5%)	1637(72.6%)	
Tumor size			0.952
≤ 2 cm	88 ( 3.9%)	86 ( 3.8%)	
2-4 cm	961 (42.6%)	949 (42.1%)	
> 4 cm	957 (42.4%)	960 (42.6%)	
Unknown	249 (11.0%)	260 (11.5%)	
Nstage			0.758
N0	1245 (55.2%)	1254 (55.6%)	
N1	852 (37.8%)	833 (36.9%)	
Nx	158 ( 7.0%)	168 ( 7.5%)	

8140/3, adenocarcinoma; 8500/3, infiltrating duct carcinoma; 8480/3, mucinous adenocarcinoma; 8246/3, neuroendocrine carcinoma

**Fig. 4:**
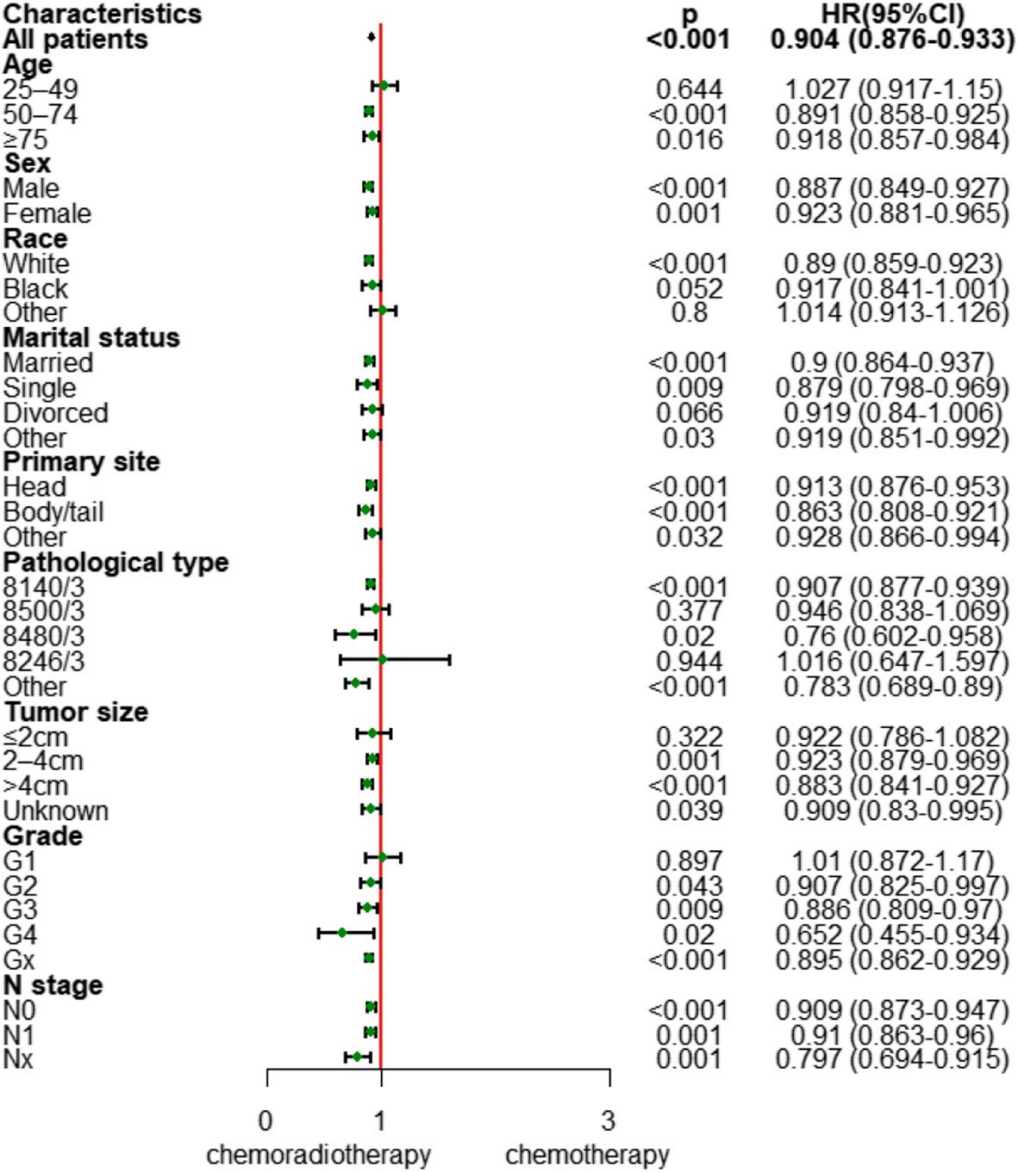
Forest plot based on hazard ratios after propensity score matching

**Fig. 5:**
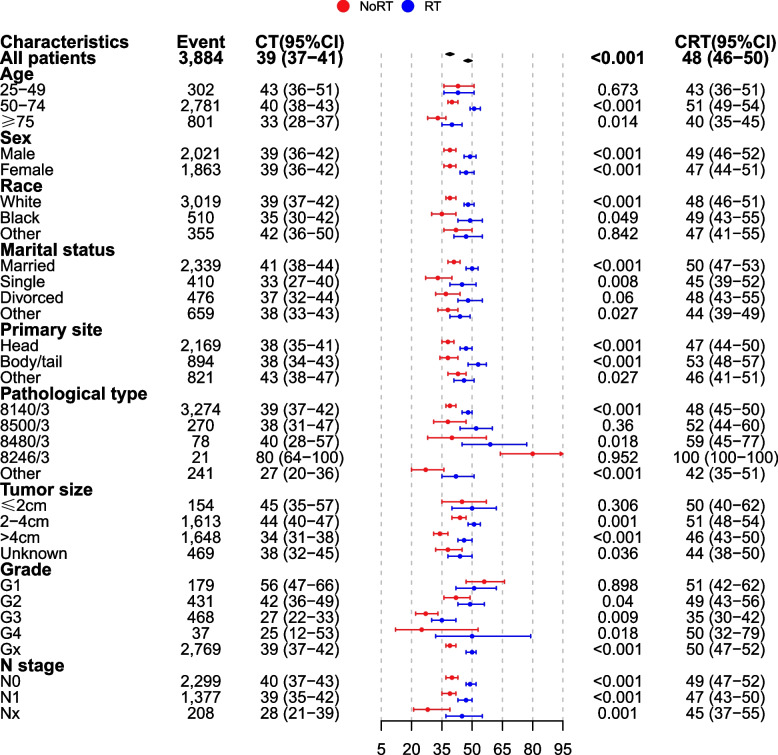
Forest plot based on overall survival rates after propensity score matching

As shown in Fig. 4 and Fig. 5, in the subgroup analysis, regardless of sex, N stage, or primary site, chemoradiotherapy significantly reduced the risk of death and improved survival.

For age, marital status, grade, and tumor size, all other subgroups had significantly improved survival with chemoradiotherapy, except for the age < 50 years (HR, 1.027; 95% CI, 0.917–1.15; *p* = 0.644), divorced (HR, 0.919; 95% CI, 0.84–1.006; *p* = 0.066), grade 1 (HR, 1.01; 95% CI, 0.872–1.17; *p* = 0.897) and tumor ≤ 2 cm (HR, 0.322; 95% CI, 0.786–1.082; *p* = 0.322) subgroups. In terms of pathological types, adenocarcinoma (HR, 0.907; 95% CI, 0.877–0.939; *p* < 0.001), mucinous adenocarcinoma (HR, 0.76; 95% CI, 0.602–0.958; *p* = 0.02) and “other” types (HR, 0.783; 95% CI, 0.689–0.89; *p* < 0.001) could benefit significantly from chemoradiotherapy, whereas infiltrating duct carcinoma (HR, 0.946; 95% CI, 0.838–1.069; *p* = 0.377) and neuroendocrine carcinoma (HR, 1.016; 95% CI, 0.647–1.597; *p* = 0.944) did not benefit from chemoradiotherapy. White race (HR, 0.89; 95% CI, 0.859–0.923; *p* < 0.001) had significant benefits from chemoradiotherapy, while “other” races (HR, 1.014; 95% CI, 0.913–1.126; *p* = 0.8) did not, and Black race (HR, 0.917; 95% CI, 0.841–1.001; *p* = 0.52) had critical benefits in the HR-based subgroup analysis and significant benefits in the survival rate-based subgroup analysis (49% vs. 35%, *p* = 0.049).

## Discussion

Previous randomized controlled trials have reported inconsistent results regarding the effect of chemoradiotherapy on unresectable locally advanced pancreatic cancer. Of the 5 retrieved randomized controlled trials, 2 found survival benefits of chemoradiotherapy on ULAPC, while 3 failed to identify any survival benefits. The earliest related randomized controlled trial, which dates back to 1985, compared 5-fluorouracil (5-FU) alone with 5-FU plus radiotherapy models [9]. Overall survival was not improved in the chemoradiotherapy group (median OS: 8.2 vs. 8.3 months). The research of the Gastrointestinal Tumor Study Group [10] in 1988 compared streptozocin, mitomycin C, and 5-FU (SMF) with SMF plus radiotherapy. The overall survival at 1 year in the chemoradiotherapy and chemotherapy groups was 41% and 19% (*p* < 0.02), respectively. In 2008, Chauffert et al. [11]. investigated 119 patients who were randomly assigned to either the CRT arm [radiotherapy plus cisplatin and 5-FU, followed by maintenance gemcitabine (GEM)] or the GEM arm, and the survival time of the CRT arm was even shorter than that of the GEM arm (median OS: 8.6 vs. 13 months, *p* = 0.03). In a trial of the Eastern Cooperative Oncology Group [12], 74 patients were randomly divided into GEM plus radiotherapy and GEM groups. In the CRT group, the survival time was significantly prolonged (median OS: 11.1 vs. 9.2 months, *p* = 0.017). The LAP07 clinical trial [13] evaluated the effect of chemoradiotherapy (radiotherapy plus capecitabine) vs. chemotherapy on survival in patients after 4 months of gemcitabine with or without erlotinib, and the results showed no significant difference in overall survival between the two groups (median OS: 15.2 vs. 16.5 months, *p* = 0.83).

Our study overcomes the limitations of small randomized controlled trials and further confirms the role of chemoradiotherapy in a large cohort of 5002 patients. In our study, multivariate analysis showed that radiotherapy was an independent prognostic factor for ULAPC. The median overall survival was two months longer (12 vs. 10 months) in the chemoradiotherapy group than in the chemotherapy group, both before and after propensity score matching. Therefore, chemotherapy combined with radiotherapy is strongly recommended for ULAPC patients to improve survival.

Pancreatic cancer most commonly occurs in the head, followed by the body and tail, which have been shown to have shorter survival [15]. In our study, more than half of the patients (55.1%) had pancreatic cancer in the head, but no difference in survival was found between the two. Pancreatic adenocarcinoma represents most primary pancreatic cancers [16]. In this study, adenocarcinoma accounted for 82.7%, while other rare subtypes, such as mucinous adenocarcinoma and neuroendocrine carcinoma, accounted for 2.1% and 0.8%, respectively. However, univariate analysis found no difference in survival between different pathological types. Our study demonstrated that older age (≥ 75 years), single, larger tumors (> 4 cm) and local lymph node invasion were significantly associated with poorer survival in both univariate and multivariate analyses, which were in accordance with previously published findings [17, 18].

Our work also further analyzed the specific beneficiaries of chemoradiotherapy. Subgroup analysis suggested that chemoradiotherapy significantly reduced the risk of death regardless of sex, N stage, or primary site. For age, marital status, grade and tumor size, the following subgroups benefited significantly from chemoradiotherapy: age ≥ 50 years, not divorced, grade 2–4, tumor size > 2 cm, adenocarcinoma, mucinous adenocarcinoma and white race. These results can help us screen out the individuals who are suitable for chemoradiotherapy in clinical practice.

In recent years, due to advances in radiation technology and the improvement of chemotherapy regimens, the survival time of ULAPC patients receiving chemoradiotherapy has also improved [19–24]. However, due to the lack of information about chemotherapy regimens, radiotherapy doses, irradiation techniques and tumor marker like CA19-9 in the SEER database, we cannot assess the impact of these factors on survival. Second, since the current data of the SEER database use AJCC 6/7th staging, the stage III patients in our study were stage IIIA patients in the AJCC 8th staging [25], and the impact of the specific number of lymph nodes on survival cannot be further analyzed. Third, since our study is a single-arm retrospective study, there may be selectivity bias in the process of data collection. Therefore, more prospective studies and comparative analyses need to be performed in the future.

## Conclusions

In summary, our study retrospectively analyzed patients with unresectable locally advanced pancreatic cancer from 2004 to 2016 in the SEER database. We confirmed the survival benefits of chemoradiotherapy for unresectable locally advanced pancreatic cancer patients according to the results of univariate and multivariate Cox regression analysis and propensity score matching analysis. We also provided a detailed description of the beneficiaries of chemoradiotherapy, which is of guiding significance for clinical work.

## Supplementary Information

**Additional file 1: Fig. S1.** Forest plot based on hazard ratios before propensity score matching. 

**Additional file 2: Fig. S2.** Forest plot based on overall survival rates before propensity score matching.

## Data Availability

Data in this paper are available from the Surveillance, Epidemiology, and End Results (SEER) Program (https://seer.cancer.gov/). Detailed data can be obtained by following the steps in the flowchart of the method section.
