# Supplementary figures and images for: Impact of chemoradiotherapy on the survival of unresectable locally advanced pancreatic cancer: a retrospective cohort analysis
Source: BMC Gastroenterol. 2023 Apr 5;23:107. doi: 10.1186/s12876-023-02739-x (PMC10077630; doi:10.1186/s12876-023-02739-x)

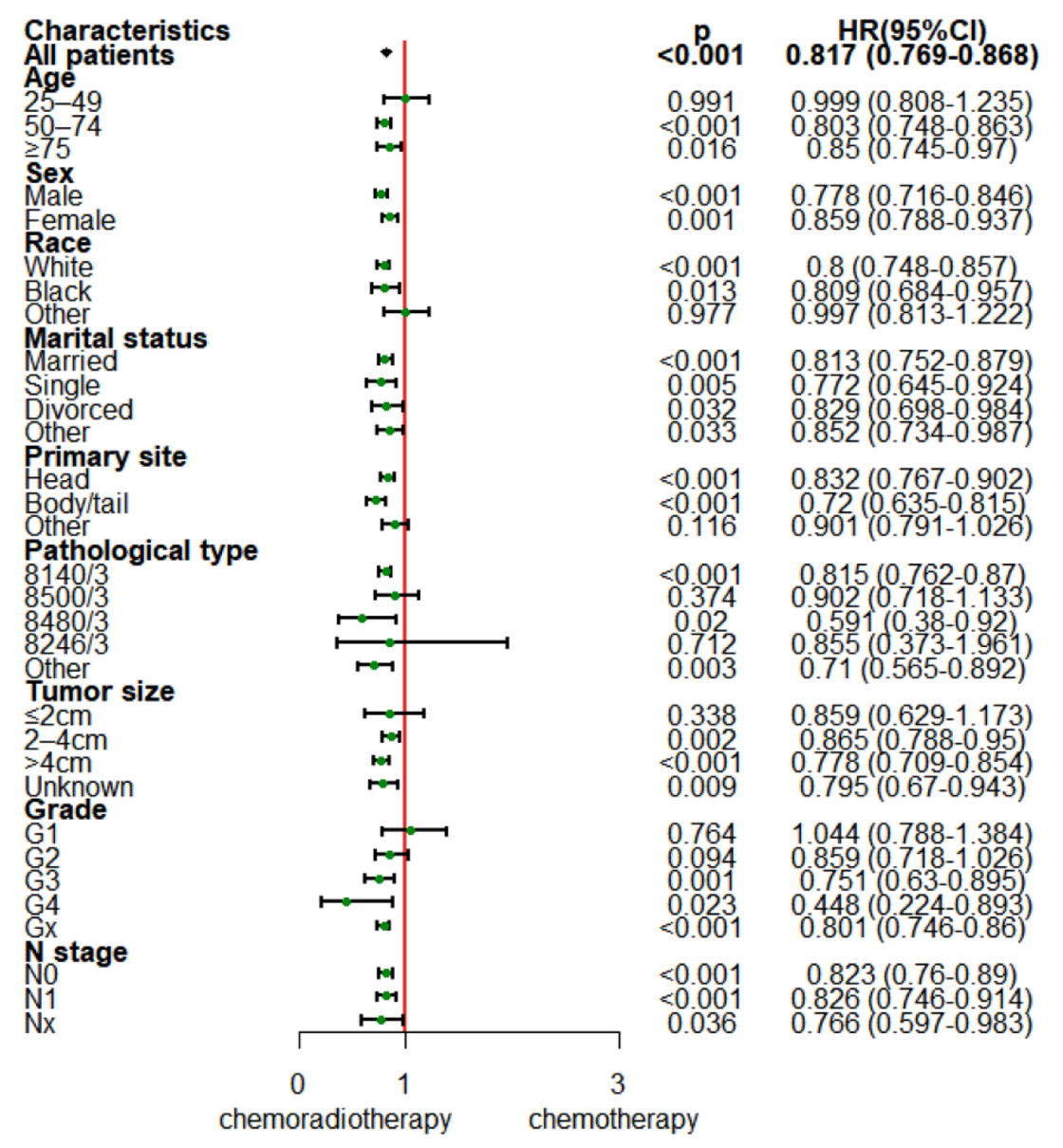


**Fig. S1** Forest plot based on hazard ratios before propensity score matching.

Supplement: Supplementary file 1 — Additional file 1: Fig. S1. Forest plot based on hazard ratios before propensity score matching. [file 12876_2023_2739_MOESM1_ESM.docx]

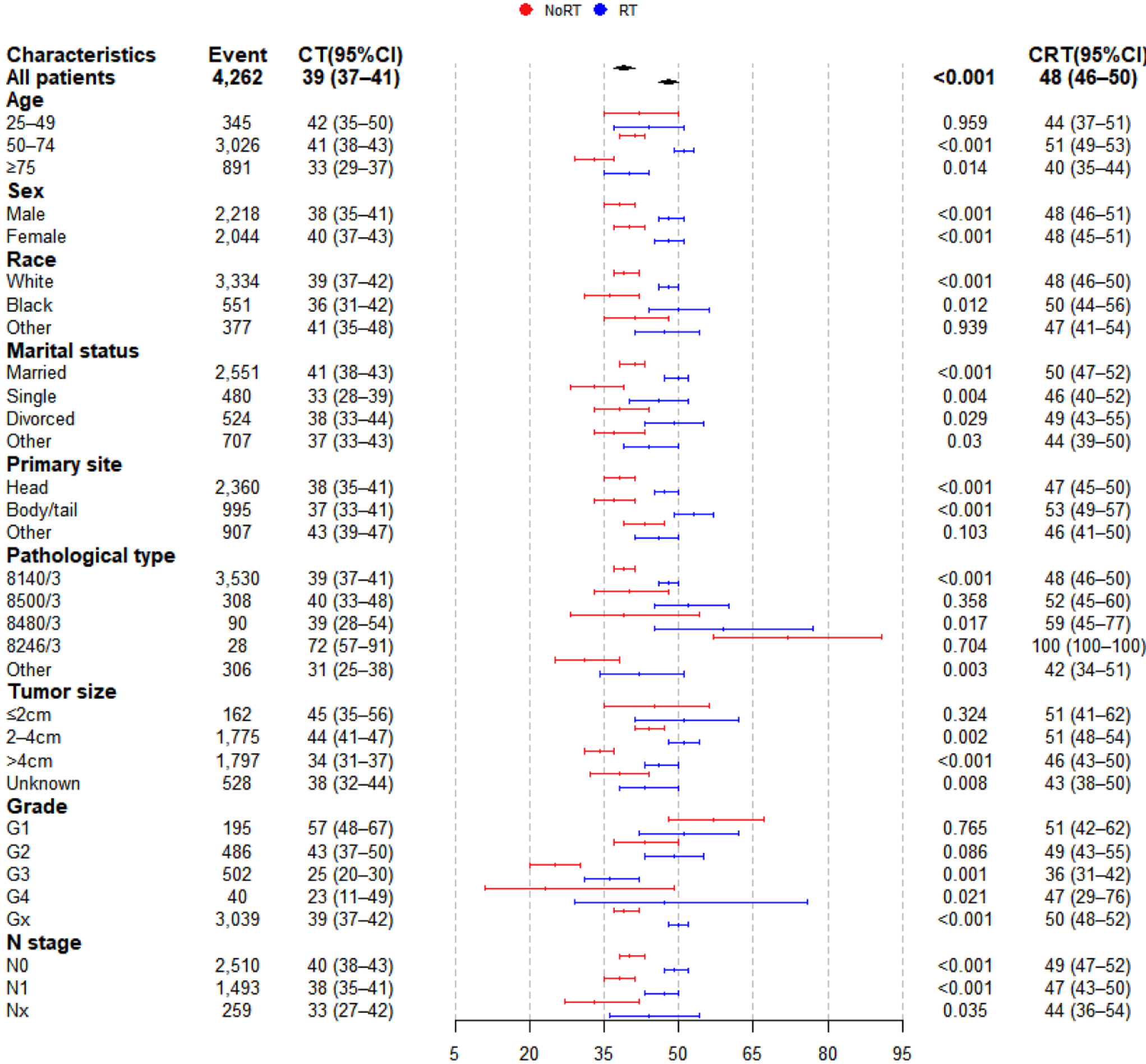


**Fig. S2** Forest plot based on overall survival rates before propensity score matching.

Supplement: Supplementary file 2 — Additional file 2: Fig. S2. Forest plot based on overall survival rates before propensity score matching. [file 12876_2023_2739_MOESM2_ESM.docx]
